# The prevalence and health burden of malnutrition in Belgian older people in the community or residing in nursing homes: results of the NutriAction II study

**DOI:** 10.1007/s40520-018-0957-2

**Published:** 2018-04-30

**Authors:** Maurits F. J. Vandewoude, Janneke P. van Wijngaarden, Lieven De Maesschalck, Yvette C. Luiking, André Van Gossum

**Affiliations:** 1 Department of Geriatrics (ZNA), University of Antwerp, Antwerp, Belgium; 2 Nutricia Research, Nutricia Advanced Medical Nutrition, Utrecht, The Netherlands; 3 Mobilab, Thomas More University College, Geel, Belgium; 4 Nutrition Support Team, Department of Gastroenterology, Hôpital Erasme, ULB, Brussels, Belgium

**Keywords:** Malnutrition, MNA-SF, Mobility, ADL, Community, Nursing home

## Abstract

**Introduction:**

In 2008, the NutriAction study showed that (risk of) malnutrition was highly prevalent (57%) among Belgian older people living in the community or in a nursing home. In 2013, this study was repeated to re-evaluate the occurrence of malnutrition, as well as mobility problems and dependence in activities of daily living (ADL).

**Methods:**

Health care professionals (HCPs) associated with homecare organizations and nursing homes across Belgium were invited to screen their patients and complete an online questionnaire. Nutritional status, presence of pre-specified comorbidities, mobility, and ADL dependency were assessed.

**Results:**

In total, 3299 older patients were analysed: 2480 (86.3 ± 6.3 years) nursing home (NH) residents and 819 (82.7 ± 6.1 years) community dwelling (CD). Overall, 12% was malnourished (MNA-SF score < 8) and 44% was at risk of malnutrition (MNA-SF 8–11). The highest prevalence of (risk of) malnutrition was observed in NHs (63%) and in patients with dementia (CD: 68%; NH: 82%) or depression (CD: 68%; NH: 79%). Of all malnourished individuals, 49% was recognized as malnourished by HCPs and 13% of the malnourished recognized themselves as such. Mobility (stair climbing and walking) and ADL dependency (Belgian KATZ score) were impaired in older people with (risk of) malnutrition in comparison with individuals with normal nutritional status (*p* < 0.001).

**Discussion:**

Despite public awareness initiatives, the prevalence of malnutrition remained stable among Belgian older people seen by HCPs in the period 2008–2013. Moreover, malnutrition is not well recognized.

**Conclusion:**

Under-recognition of malnutrition is problematic, because associated loss of mobility and independence may accelerate the transformation of frailty into disability in older people.

**Electronic supplementary material:**

The online version of this article (10.1007/s40520-018-0957-2) contains supplementary material, which is available to authorized users.

## Introduction

Older people show benefit from an optimal nutritional status, which sustains lean body mass, especially muscle; this in turn supports mobility, resilience, and resistance to potential disease. However, the risk of malnutrition increases with aging due to the presence of (multiple) chronic diseases, dementia, depression, and age-related anorexia caused by loss of appetite, swallowing difficulties, and alterations in taste sensibility [1–4]. Malnutrition is a problem, because it renders older people more vulnerable to (long term) hospitalisation, medical complications, and because it has a negative impact on their quality of life [1, 5]. Moreover, malnourished older people are likely to die sooner than their well-nourished peers [6–8].

A meta-analysis on the prevalence of malnutrition (Mini Nutritional Assessment (MNA) score < 17) in older adults showed that 3% of community dwelling older adults were malnourished [3]. Prevalence increased with level of care: out-patients (6%), older adults receiving homecare (9%), hospitalized patients (22%), nursing home residents (18%), and patients in long-term care (29%) [3]. The prevalence of risk of malnutrition (MNA: 17-23.5) was 27% in community dwelling older people, 31% in out-patients, 48% in older patients receiving homecare, 46% in hospitalized older people, 48% in nursing home residents, and 49% in older patients receiving long-term care. Meta-regression analysis of the studies furthermore showed that (risk of) malnutrition was directly associated with the setting-related level of dependence [3].

The notion that disease-related malnutrition is a problem in industrialized countries has caught the attention of policy makers. In 2003 the European Council issued a resolution appendix with recommendations regarding assessment of nutritional status and nutritional care in the hospital setting [9]. However, especially, in case of older people, malnutrition often exists prior to admission and persists after discharge. In 2007, the Belgian National Food and Health Plan organised a forum with key opinion leaders from society, academia, and authorities, who developed an action plan with recommendations on nutrition care for older adults with homecare or residing in care homes [10]. In line with this, in 2008, a national screening—NutriAction—was organised to assess risk and prevalence of malnutrition in community dwelling older adults and older nursing home residents in Belgium, and to increase health care professionals’ awareness of malnutrition screening [11]. This screening showed that among Belgian older people, community dwelling, or in a nursing home, (risk of) malnutrition was highly prevalent (57%; MNA-SF ≤ 11), and that 16% had a BMI < 20 kg/m^2^ [12]. This is in agreement with percentages observed by Cereda et al. [3], and with the percentage of malnourished nursing home residents in Austria and Germany, as observed in the NutritionDay database, an initiative of the European Society for clinical nutrition and metabolism (ESPEN) [13].

In 2013, the Belgian NutriAction screening was repeated to re-evaluate the prevalence of malnutrition 5 years after the first screening and to characterize patients with (risk of) malnutrition according to care setting and comorbidities. In addition, the ability of health care professionals to recognize malnutrition and the health burden of malnutrition on mobility and dependence in activities of daily living was assessed. Another goal was to reinforce awareness of the existence of malnutrition and the need to treat it among involved health care professionals.

## Methods

### Design and participants

For this cross-sectional study (NutriAction II), general practitioners (GPs) and nurses associated with homecare organizations for community dwelling patients, and HCPs in nursing homes in Belgium were approached to assess the nutritional status of people aged 70 years and older under their care, from February until April 2013. There were no exclusion criteria. GPs, nurses and other HCPs received a standardised instruction on how to complete the online questionnaire developed for this screening.

The NutriAction II project was approved by the ethical committee of the University Hospital of Antwerp and has been performed in accordance with the ethical standards as laid down in the 1964 Declaration of Helsinki and its later amendments or comparable ethical standards.

The privacy of participants was guaranteed according to Belgian legislation by anonymising the data. Informed consent was obtained from all individual participants included in the study.

### Questionnaire

Nurses, GPs, and other HCPs responsible for the care of the participants at home or in nursing homes performed the screening and completed the online questionnaire. For NutriAction II, the 2008 questionnaire [12] was slightly adapted. In both the 2008 and 2013 questionnaires, gender, age, height, and weight (estimated/determined) were inquired. Furthermore, the use of nutritional supplementation and number of meals used per day was documented. Both questionnaires also contained the complete ‘short form Mini Nutrition Assessment’ questionnaire (MNA-SF), but in 2008, participants scored ‘at risk of malnutrition’ when the MNA-SF score was 11 or lower, whereas the 2013 questionnaire used two cut-off points: normal nutritional status (MNA-SF ≥ 12) and malnutrition (MNA-SF score < 8). Risk of malnutrition was in between those cut-off points (8–11). The 2013 questionnaire was more extensive. Regarding general health status, the 2008, questionnaire contained six questions that could be answered with ‘yes’ or ‘no’, concerning diabetes, cancer, swallowing disorders due to Parkinson and stroke, functional impairment due to stroke, and wounds/decubitus. The 2013 questionnaire contained pre-specified comorbidities and functional impairments that could be checked.

To establish the ability to recognize malnutrition, the HCP was asked prior to nutritional assessment if he or she considered the older person under assessment malnourished. The person assessed was also asked if he or she considered his- or herself malnourished. Mobility was assessed as the ability of the patient to climb stairs (15 steps) and to walk outside for 5 min without taking a rest (yes/no). Independence in Activities of Daily Living (ADL) was assessed using the Belgian Katz ADL questionnaire with the familiar physical functions (bathing, dressing, transferring, toileting, continence, and feeding) and two additional cognitive functions (orientation in time and space) [14]. The Belgian Katz score builds from category O, representing full independence, via categories A, B, and C, representing progressing physical dependency. When disorientation in time and space (incipient dementia) was present, participants were scored as D (= demented), or C_D (full physical dependency with incontinence in people with dementia).

The 2013 questionnaire furthermore contained questions about number and type of medications used, recent hospitalisations (< 3 months), and could be used to compare screening instruments for (risk of) malnutrition. However, these items are outside the scope of this paper, see for all details the online resource containing the full Dutch 2013 questionnaire (ESM_1).

### Statistics

The prevalence of malnutrition and risk of malnutrition were reported in the different care settings (nursing homes and community dwelling). The distribution of nutritional status (malnourished, at risk of malnutrition or normal nutritional status according to MNA-SF) was also reported within a selection of medical conditions. Continuous variables were expressed as mean ± SD, proportions were expressed as n (%). For continuous variables, differences between groups were analysed using the Student *t* test, or ANOVA, when three groups were compared. The Chi-square test was used for categorical variables. Statistical significance was considered for *p* values < 0.05. All analyses were performed in SPSS (IBM SPSS Statistics Version 19).

## Results

### Characteristics of assessed population

Nurses, GPs, and other HCPs associated with eight homecare organizations and from 36 nursing homes, participated in the study. This sample is representative for older people in Belgium. A total of 3641 persons were screened; 342 individuals were excluded, based on age < 70 years or on double entry. This left 3299 older people for analysis, of whom 819 (25%) were community dwelling (CD) and 2480 (75%) were nursing home residents (NH). The majority of the population was female (76%). The mean age was 85.4 ± 6.4 years. Characteristics are shown per setting, i.e., NH or CD, in Table 1. The results categorized by nutritional status (MNA-SF) are shown separately for the CD (Table 2) and NH population (Table 3).

**Table 1:** Characteristics of the nursing home and community dwelling study populations

Parameter	Nursing home (*n* = 2480)	Community dwelling (*n* = 819)	*p* value
Age (years) mean ± SD	86.3 ± 6.2	82.7 ± 6.1	< 0.001
Sex (% male/female)	22/78	32/68	< 0.001
Presence of comorbidities			
Cancer	171 (7%)	87 (11%)	0.001
Chronic heart failure	285 (12%)	113 (14%)	0.079
COPD	141 (6%)	50 (6%)	0.656
Dementia	1074 (43%)	113 (14%)	< 0.001
Depression	367 (15%)	47 (6%)	< 0.001
Diabetes	440 (18%)	163 (20%)	0.165
Fractures	313 (13%)	63 (8%)	< 0.001
Parkinson	140 (6%)	37 (5%)	0.214
Rheumatoid arthritis	122 (5%)	116(14%)	< 0.001
Stroke	284 (12%)	59 (7%)	0.001
Number of comorbidities mean ± SD	1.5 ± 1.1	1.1 ± 0.98	< 0.001
0	407 (16%)	208 (25%)	
1	1035 (42%)	391 (48%)	
2	647 (26%)	147 (18%)	
3	282 (11%)	55 (7%)	
4	85 (3%)	10 (1%)	
5	18 (0.7%)	7 (0.9%)	
6	6 (0.2%)	1 (0.1%)	
BMI (kg/m^2^) ± SD	24.3 ± 5.4	26.3 ± 5.3	< 0.001
Nutritional status (MNA-SF)			< 0.001
% malnourished	336 (14%)	56 (7%)	
% at risk of malnutrition	1214 (49%)	241 (29%)	
% normal	907 (37%)	519 (63%)	
Weight loss last 3 months			< 0.001
No weight loss	1541 (62%)	601 (73%)	
1–3 kg	474 (19%)	88 (11%)	
3–6 kg	143 (6%)	37 (5%)	
> 6 kg	47 (2%)	28 (3%)	
Unknown	275 (11%)	65 (8%)	
Use of ONS	204 (8%)	14 (2%)	< 0.001
Being able to climb stairs	391 (16%)	268 (33%)	< 0.001
Being able to walk outside for 5 min	853 (34%)	425 (52%)	< 0.001
Katz ADL score			< 0.001
Cat O	460 (19%)	228 (28%)	
Cat A	346 (14%)	214 (26%)	
Cat B	242 (10%)	188 (23%)	
Cat C	327 (13%)	129 (16%)	
Cat D (D = demented)	242 (10%)	5 (0.6%)	
Cat C_D (fully dependent)	863 (35%)	55 (7%)	

Data are presented as *n* (%), except for age, sex, number of comorbidities, and BMI. Participants had no more than 6 comorbidities. Nutritional status was missing in *n* = 26 (CD: *n* = 3, NH: *n* = 23)

*ADL* activities of daily living, *BMI* body mass index, *COPD* chronic obstructive pulmonary disease, *MNA-SF* mini nutritional assessment—short form, *ONS* oral nutritional supplement

**Table 2:** Characteristics of community dwelling study population (*n* = 819), by nutritional status (based on MNA-SF)

Parameter	Malnourished (*n* = 56, 7%)	At risk of malnutrition (*n* = 241, 29%)	Normal nutritional status (*n* = 519, 63%)	*p* value
Age (years) mean ± SD	83.0 ± 6.0	83.4 ± 6.0	82.3 ± 6.1	0.063
Sex (% male/female)	32/68	29/71	34/66	0.376
Number of comorbidities mean ± SD	1.4 ± 0.9	1.4 ± 1.0	1.0 ± 0.9	< 0.001
BMI (kg/m^2^) mean ± SD	20.2 ± 3.5	24.4 ± 5.1	27.9 ± 4.8	< 0.001
Weight loss last 3 months				< 0.001
No weight loss	3 (5%)	119 (49%)	476 (92%)	
1–3 kg	9 (16%)	47 (20%)	32 (6%)	
3–6 kg	18 (32%)	19 (8%)	0	
> 6 kg	15 (27%)	13 (5%)	0	
Unknown	11 (20%)	43 (18%)	11 (2%)	
Use of ONS	4 (7%)	8(3%)	2 (0.4%)	< 0.001
Being able to climb stairs	10 (18%)	49 (20%)	208 (40%)	< 0.001
Being able to walk outside for 5 min	19 (34%)	97 (40%)	306 (59%)	< 0.001
Katz ADL score				< 0.001
Cat O	6 (11%)	36 (15%)	186 (36%)	
Cat A	11 (20%)	50 (21%)	153 (30%)	
Cat B	14 (25%)	71 (30%)	101 (20%)	
Cat C	13 (23%)	53 (22%)	63 (12%)	
Cat D (D = demented)	0	1 (0.4%)	4 (1%)	
Cat C_D (fully dependent)	12 (21%)	30 (12%)	12 (2%)	

Data are presented as *n* (%), except for age, sex, number of comorbidities, and BMI. Nutritional status was missing in *n* = 3; results may therefore not add up to 100%

*ADL* activities of daily living, *BMI* body mass index, *MNA-SF* mini nutritional assessment—short form, *ONS* oral nutritional supplement

Individuals in the NH population had on average more comorbidities than the CD individuals (Table 1). In the NH population, the individual number of comorbidities was higher with worsening of nutritional status (Table 3). Although this was less obvious in the CD population, individuals with (risk of) malnutrition had on average more comorbidities than the well-nourished (Table 2). The neuropsychological comorbidities depression and/or dementia were most common (49% of the total population), and more prevalent in the NH than in the CD population (Table 1).

### Nutritional status

#### Prevalence of (risk of) malnutrition

The prevalence of (risk of) malnutrition in the total study population was 56% (12% malnourished and 44% at risk). The prevalence of (risk of) malnutrition was higher in NH residents compared to CD individuals (63% vs. 36%, *p* < 0.001; Table 1). The highest prevalence of (risk of) malnutrition was observed in patients with dementia (CD: 68%; NH: 82%), and depression (CD: 68%; NH: 79%; Table 4). In nursing homes, malnutrition (risk) was also high among patients with Parkinson’s disease (72%). Furthermore, the prevalence of (risk of) malnutrition was 47% in CD cancer patients and 60% in NH patients with cancer (Table 4).

**Table 3:** Characteristics of the nursing home study population (*n* = 2480), by nutritional status (based on MNA-SF)

Parameter	Malnourished (*n* = 336, 14%)	At risk of malnutrition (*n* = 1214, 49%)	Normal nutritional status (*n* = 907, 37%)	*p* value
Age (years) mean ± SD	86.7 ± 6.4	86.7 ± 6.2	85.6 ± 6.2	< 0.001
Sex (% male/female)	22/78	20/80	23/77	0.258
Number of comorbidities mean ± SD	1.9 ± 1.2	1.6 ± 1.1	1.1 ± 1.0	< 0.001
BMI (kg/m^2^) ± SD	19.7 (3.9)	23.2 (4.8)	27.4 (4.7)	< 0.001
Weight loss last 3 months				< 0.001
No weight loss	34 (10%)	679 (56%)	817 (90%)	
1–3 kg	85 (25%)	307 (25%)	77 (9%)	
3–6 kg	101 (30%)	39 (3%)	0	
> 6 kg	39 (12%)	8 (1%)	0	
Unknown	77 (23%)	181 (15%)	13 (1%)	
Use of ONS	73 (22%)	111 (9%)	19 (2%)	< 0.001
Being able to climb stairs	10 (3%)	130 (11%)	246 (27%)	< 0.001
Being able to walk outside for 5 min	36 (11%)	310 (26%)	496 (55%)	< 0.001
Katz ADL score				< 0.001
Cat O	13 (4%)	133 (11%)	312 (34%)	
Cat A	13 (4%)	113 (9%)	219 (24%)	
Cat B	31 (9%)	126 (10%)	85 (9%)	
Cat C	58 (17%)	175 (14%)	92 (10%)	
Cat D (D = demented)	15 (5%)	112 (9%)	109 (12%)	
Cat C_D (fully dependent)	206 (61%)	555 (46%)	90 (10%)	

Data are presented as *n* (%), except for age, sex, number of comorbidities, and BMI. Nutritional status is missing in *n* = 23; results may therefore not add up to 100%

*ADL* activities of daily living, *BMI* body mass index, *MNA-SF* mini nutritional assessment—short form, *ONS* oral nutritional supplement

**Table 4:** Nutritional status determined with MNA-SF of community dwelling older adults and nursing home residents, by their comorbidities

	Malnourished	At risk of malnutrition	Normal nutritional status
Community dwelling (*n* = 816)	*n* = 56 (7%)	*n* = 241 (29%)	*n* = 519 (63%)
Presence of comorbidities			
Cancer (*n* = 87)	8 (9%)	33 (38%)	46 (53%)
Chronic heart failure (*n* = 113)	6 (5%)	41 (36%)	66 (58%)
COPD (*n* = 50)	8 (16%)	16 (32%)	26 (52%)
Dementia (*n* = 110)	16 (15%)	59 (54%)	35 (32%)
Depression (*n* = 44)	8 (18%)	22 (50%)	14 (32%)
Diabetes (*n* = 162)	5 (3%)	44 (27%)	113 (70%)
Fractures (*n* = 62)	4 (7%)	24 (39%)	34 (55%)
Parkinson (*n* = 37)	3 (8%)	18 (49%)	16 (43%)
Rheumatoid arthritis (*n* = 116)	5 (4%)	40 (35%)	71 (61%)
Stroke (*n* = 59)	4 (7%)	18 (31%)	37 (63%)
Nursing home (*n* = 2457)	*n* = 336 (14%)	*n* = 1214 (49%)	*n* = 907 (37%)
Presence of comorbidities			
Cancer (*n* = 169)	28 (17%)	74 (44%)	67 (40%)
Chronic heart failure (*n* = 282)	32 (11%)	150 (53%)	100 (36%)
COPD (*n* = 141)	25 (18%)	67 (48%)	49 (35%)
Dementia (*n* = 1051)	212 (20%)	649 (62%)	190 (18%)
Depression (*n* = 344)	81 (24%)	192 (56%)	71 (21%)
Diabetes (*n* = 437)	49 (11%)	208 (47%)	180 (41%)
Fractures (*n* = 310)	58 (19%)	146 (47%)	106 (34%)
Parkinson (*n* = 139)	29 (21%)	71 (51%)	39 (28%)
Rheumatoid arthritis (*n* = 122)	20 (16%)	59 (48%)	43 (35%)
Stroke (*n* = 284)	38 (13%)	147 (52%)	99 (35%)

Data are presented as *n* (%). Data represent nutritional status categorized per comorbidity (row percentage). The list of scored comorbidities was longer, reported comorbidities represent comorbidities with prevalence > 5%. Numbers do not add up because comorbidities overlap (column totals)

*COPD* chronic obstructive pulmonary disease

No *p* values are presented in Table 4, because comorbidities overlap and combinations may affect nutritional status differently than single comorbidities. As such, Table 4 provides only an indication of the prevalence of (risk of) malnutrition presented for single comorbidities.

#### Recognition of malnutrition

**Fig. 1:**
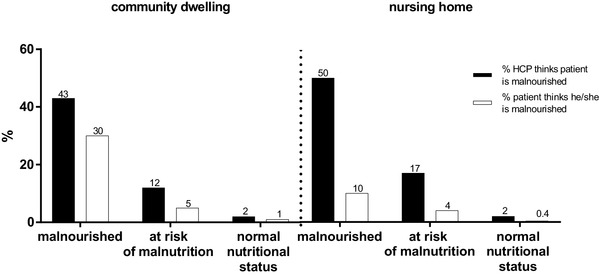
Recognition of malnutrition (based on MNA-SF) in % by health care professionals (HCP; solid bars) and patients themselves (white bars) in the community (left) and in nursing homes (right)

Prior to nutritional assessment, only half (49%) of the malnourished patients in the total study population were recognized as malnourished by the HCPs. This percentage was similar for CD and NH older individuals (*p* = 0.343; Fig. 1). Only 13% of the malnourished patients indicated themselves as being malnourished. This percentage was lower in the NH than the CD population (Fig. 1; *p* < 0.001).

#### Use of ONS

Seven per cent of the included older people used an oral nutritional supplement (ONS). The use of ONS was more common among the NH than the CD population (Table 1). Only 20% of the malnourished older people and 8% of those at risk of malnutrition in the total study population used ONS. Fewer CD malnourished older people than malnourished NH residents used ONS (Table 2, 3; *p* = 0.011).

### Mobility and ADL

Of the total population, 20% was able to climb stairs. This percentage was higher in the CD than in the NH population (Table 1). Of the total population, 39% of the population was able to walk outside for 5 min. This percentage was also higher in the CD than the NH population (Table 1). In the total study population, 95% of those with malnutrition and 88% of those at risk of malnutrition, could not climb stairs, vs. 68% of the people with a normal nutritional status (*p* < 0.001). Of the CD population, malnourished older adults were less able to climb stairs than those with normal nutritional status (Table 2). The same was observed among NH residents, where the contrast was even larger (Table 3). In the total study population, 86% of those with malnutrition and 72% of those at risk of malnutrition could not walk for 5 min without interruption, vs. 44% of the people with a normal nutritional status (*p* < 0.001). Of the CD population, older adults with malnutrition were less able to walk for 5 min without interruption, than those with normal nutritional status (Table 2). The same was observed among NH residents, and again the contrast was larger (Table 3).

Of the total population, 21% was independent in ADL (Katz = O). Nursing home residents were more dependent in ADL than the community dwelling older adults (Table 1). A worse ADL score (Katz scores D and C_D) was observed in individuals with (risk of) malnutrition in comparison with individuals with normal nutritional status, and only a few older adults with (risk of) malnutrition were independent in ADL (Katz = O) (overall: 10%, CD: 14%, NH: 9%). Of normal nourished individuals, 36% of the CD and 34% of the NH individuals were ADL independent. (Table 2 and 3). Furthermore, ADL status varied with comorbidities (Supplemental Table 1a and 1b in ESM_2), with the highest ADL dependency in dementia patients.

## Discussion

The overall prevalence of (risk of) malnutrition in older individuals with homecare or residing in nursing homes, determined with the MNA-SF (cut-off ≤ 11), was similar (56%) to the observed prevalence with the previous NutriAction screening in 2008 (57%) [12]. The observed prevalence of malnutrition was within the range of observations by others [3, 13], although the prevalence of (risk of) malnutrition in our community dwelling population was slightly higher [3]. This may be explained by the selection of community dwelling individuals that were already under the care of a HCP. The 2013 NutriAction study furthermore revealed that the prevalence of (risk of) malnutrition was almost twice as high in the nursing home population (63%), compared to the community dwelling population (36%).

In general, comorbidities were more prevalent in nursing homes than in the community, and were higher with worsening of nutritional status. We speculate that malnourished individuals with more comorbidities are less able to live independently and, therefore, are more prevalent in the nursing home. Of the total population, 49% suffered from the neuropsychological comorbidities depression and/or dementia. In nursing homes, this was 58%, compared to 20% in community dwelling older individuals. In demented or depressed older people, the prevalence of (risk of) malnutrition seems higher than for other comorbidities. This was observed in both settings (community and nursing home). This observation supports the finding by Valentini et al. [13] that the presence of dementia increased the odds of being malnourished or at risk of malnutrition (BMI < 22) in nursing home residents of 50 years and older. Results from the Longitudinal Aging Study Amsterdam (LASA) suggest that depressive symptoms may actually be an important early determinant of undernutrition [4]. This could explain the higher prevalence of (risk of) malnutrition in depressed older people in our study. However, in nursing homes, with increasing dependency, the prevalence of other comorbidities rises. In this situation, dementia and depression remain major risk factors, but their individual impact on nutritional status will be diluted by the impact of other more prevalent comorbidities.

### Recognition of (risk of) malnutrition and use of ONS

Despite the high prevalence of (risk of) malnutrition, only 7% of all older individuals consumed oral nutritional supplements (ONS). In the nursing homes, only half of the malnourished and less than one-fifth of those at risk of malnutrition were recognized as such, and even less of them received ONS. This suggests that even when a low nutritional status is recognized, this not necessarily results in intervention with medical nutrition. The higher percentage of ONS-fed nursing home residents compared to community dwelling older adults, however, suggests a slightly higher awareness of health care practitioners in nursing homes of the prevalent risk of malnutrition and need for treatment. Another explanation could be that ONS is more accessible in nursing homes.

This result is less favorable than the observation by Streicher et al. [15], who used Nutrition Day data of 23,689 nursing home residents of 65 years or older, collected between 2007 and 2014, and showed that 14% of all nursing home residents (42% of those malnourished and 26% of those at risk of malnutrition) received ONS. This difference could be partly due to the lack of reimbursement of ONS in Belgium. The recognition of malnutrition among older community dwelling adults by HCPs was equally limited. The under-recognition of malnutrition by HCPs is in line with the previous observations [16, 17]. Beattie et al. [17] found that staff members of residential aged care facilities may lack sufficient knowledge on nutrition to provide responsive care, and stress the importance of enhancing nutritional awareness and assessment skills of these professionals. This was actually one of the goals of NutriAction II.

Self-awareness regarding nutritional status of older community dwellers and nursing home residents was even lower. This may be caused by the large number of neuropsychological compromised older individuals, because demented and depressed older adults may be less able to acknowledge their own nutritional status.

### Mobility and ADL

In line with findings by Cereda et al. [3], we observed that nursing home residents were more dependent in ADL than community dwelling older adults. We also observed that mobility and performance were more compromised in the malnourished, and most in the malnourished nursing home residents. In agreement with this, a worse ADL score was observed in older adults with lower nutritional status. Only 10% of the older adults who were malnourished or at risk of malnutrition was still independent. There is growing evidence of a causal relationship between an imbalanced diet, either in deficit or excess, and a reduced general functional performance of the body, particularly in older people [2]. Therefore, a low nutritional status may accelerate the transformation of frailty into disability, and eventually result in individuals exchanging their home situation for a nursing home. In addition, loss of dependency may cause further impairment of the nutritional status. This vicious circle of events is supported by the higher prevalence of dependence and malnutrition in nursing homes as observed by us and others [3].

Since a considerable percentage of those with a normal nutritional status in our study was compromised in mobility (44% could not walk 5 min) and performance (68% could not climb stairs), other factors beyond malnutrition may also play a role. Another known important contributor to functional performance, if not most important, is reduced physical activity with aging [18]. A combination of an adequate nutritional intervention and a physical activity program may improve functional performance in older people, and thus reduce the need for institutionalization. It may also improve quality of life, which may attenuate the development of depression and thereby break the vicious circle of depression as cause and consequence of malnutrition. Recommendations for nutritional support include high protein and vitamin D [19, 20].

### Strengths and limitations

The strength of this study is that a large number of older people (*n* = 3299) from both the community dwelling setting and nursing homes were included. This resulted in a representative sample of older individuals in both nursing homes and at home. In addition, this required the involvement of a variety of health care professionals, which supported the secondary aim to increase awareness among them of the existence of, and need to treat malnutrition. Another ‘strength’ is application of the MNA-SF, used in both screenings in 2008 and 2013, a well-established and validated malnutrition screening tool in older people. Furthermore, the 2013 NutriAction study yielded additional information about the prevalence of malnutrition within a variety of comorbidities. In addition, the recent NutriAction provides information about (self) recognition of malnutrition, and the relationship between nutritional status and functional performance of the included older adults.

A limitation of the study is that no information is available regarding muscle mass and strength, which makes it more difficult to determine the presence of frailty or sarcopenia. Although valuable, data regarding the prevalence of (risk of) malnutrition within comorbidities was limited to pre-specified conditions, and should be interpreted with caution, since comorbidities may overlap and as such affect nutritional status differently. Multivariate regression analysis may be necessary to unravel the contribution of different (combinations of) comorbidities to nutritional status. However, in this paper, we focused on the prevalence of malnutrition to raise awareness rather than to analyse the contribution of comorbidities to nutritional status. In addition, use of the Belgian KATZ score makes it less easy to compare ADL dependency results of this study with results of other studies. Finally, the inclusion of demented people may have complicated the evaluation of self-recognized malnutrition.

## Conclusion

The prevalence of malnutrition has remained stable among Belgian older people in the period 2008–2013. Malnutrition remains a major problem, despite all awareness initiatives by the government and nutrition-focussed organisations. Malnutrition is especially prevalent in nursing homes and in those with comorbidities such as depression and dementia. Health care professionals and patients themselves greatly underestimate malnutrition in older people, both in the community and in nursing homes. Malnutrition is associated with mobility problems and dependence in ADL, and therefore may accelerate the transformation of frailty into disability. In the future, nutritional screening could be complemented with screening for frailty and functional limitations.

## Electronic supplementary material

Below is the link to the electronic supplementary material.

Supplementary material 1 (PDF 230 KB)

Supplementary material 2 (DOCX 31 KB)
