# Correction to: The prevalence and health burden of malnutrition in Belgian older people in the community or residing in nursing homes: results of the NutriAction II study

**DOI:** 10.1007/s40520-018-0979-9

**Published:** 2018-06-12

**Authors:** Maurits F. J. Vandewoude, Janneke P. van Wijngaarden, Lieven De Maesschalck, Yvette C. Luiking, André Van Gossum

**Affiliations:** 1 Department of Geriatrics (ZNA), University of Antwerp, Antwerp, Belgium; 2 Nutricia Research, Nutricia Advanced Medical Nutrition, Utrecht, The Netherlands; 3 Mobilab, Thomas More University College, Geel, Belgium; 4 Nutrition Support Team, Department of Gastroenterology, Hôpital Erasme, ULB, Brussels, Belgium

## Correction to: Aging Clinical and Experimental Research 10.1007/s40520-018-0957-2

In the original publication, table row alignment was incorrectly formatted for all the tables. The corrected Tables 1, 2, 3 and 4 are given below.

**Table 1:** Characteristics of the nursing home and community dwelling study populations

Parameter	Nursing home (*n* = 2480)	Community dwelling (*n* = 819)	*p* value
Age (years) mean ± SD	86.3 ± 6.2	82.7 ± 6.1	< 0.001
Sex (% male/female)	22/78	32/68	< 0.001
Presence of comorbidities			
Cancer	171 (7%)	87 (11%)	0.001
Chronic heart failure	285 (12%)	113 (14%)	0.079
COPD	141 (6%)	50 (6%)	0.656
Dementia	1074 (43%)	113 (14%)	< 0.001
Depression	367 (15%)	47 (6%)	< 0.001
Diabetes	440 (18%)	163 (20%)	0.165
Fractures	313 (13%)	63 (8%)	< 0.001
Parkinson	140 (6%)	37 (5%)	0.214
Rheumatoid arthritis	122 (5%)	116(14%)	< 0.001
Stroke	284 (12%)	59 (7%)	0.001
Number of comorbidities mean ± SD	1.5 ± 1.1	1.1 ± 0.98	< 0.001
0	407 (16%)	208 (25%)	
1	1035 (42%)	391 (48%)	
2	647 (26%)	147 (18%)	
3	282 (11%)	55 (7%)	
4	85 (3%)	10 (1%)	
5	18 (0.7%)	7 (0.9%)	
6	6 (0.2%)	1 (0.1%)	
BMI (kg/m^2^) ± SD	24.3 ± 5.4	26.3 ± 5.3	< 0.001
Nutritional status (MNA-SF)			< 0.001
% malnourished	336 (14%)	56 (7%)	
% at risk of malnutrition	1214 (49%)	241 (29%)	
% normal	907 (37%)	519 (63%)	
Weight loss last 3 months			< 0.001
No weight loss	1541 (62%)	601 (73%)	
1–3 kg	474 (19%)	88 (11%)	
3–6 kg	143 (6%)	37 (5%)	
> 6 kg	47 (2%)	28 (3%)	
Unknown	275 (11%)	65 (8%)	
Use of ONS	204 (8%)	14 (2%)	< 0.001
Being able to climb stairs	391 (16%)	268 (33%)	< 0.001
Being able to walk outside for 5 min	853 (34%)	425 (52%)	< 0.001
Katz ADL score			< 0.001
Cat O	460 (19%)	228 (28%)	
Cat A	346 (14%)	214 (26%)	
Cat B	242 (10%)	188 (23%)	
Cat C	327 (13%)	129 (16%)	
Cat D (D = demented)	242 (10%)	5 (0.6%)	
Cat C_D (fully dependent)	863 (35%)	55 (7%)	

Data are presented as *n* (%), except for age, sex, number of comorbidities, and BMI. Participants had no more than 6 comorbidities. Nutritional status was missing in *n* = 26 (CD: *n* = 3, NH: *n* = 23)

*ADL* activities of daily living, *BMI* body mass index, *COPD* chronic obstructive pulmonary disease, *MNA-SF* mini nutritional assessment—short form, *ONS* oral nutritional supplement

**Table 2:** Characteristics of community dwelling study population (*n* = 819), by nutritional status (based on MNA-SF)

Parameter	Malnourished (*n* = 56, 7%)	At risk of malnutrition (*n* = 241, 29%)	Normal nutritional status (*n* = 519, 63%)	*p* value
Age (years) mean ± SD	83.0 ± 6.0	83.4 ± 6.0	82.3 ± 6.1	0.063
Sex (% male/female)	32/68	29/71	34/66	0.376
Number of comorbidities mean ± SD	1.4 ± 0.9	1.4 ± 1.0	1.0 ± 0.9	< 0.001
BMI (kg/m^2^) mean ± SD	20.2 ± 3.5	24.4 ± 5.1	27.9 ± 4.8	< 0.001
Weight loss last 3 months				< 0.001
No weight loss	3 (5%)	119 (49%)	476 (92%)	
1–3 kg	9 (16%)	47 (20%)	32 (6%)	
3–6 kg	18 (32%)	19 (8%)	0	
> 6 kg	15 (27%)	13 (5%)	0	
Unknown	11 (20%)	43 (18%)	11 (2%)	
Use of ONS	4 (7%)	8(3%)	2 (0.4%)	< 0.001
Being able to climb stairs	10 (18%)	49 (20%)	208 (40%)	< 0.001
Being able to walk outside for 5 min	19 (34%)	97 (40%)	306 (59%)	< 0.001
Katz ADL score				< 0.001
Cat O	6 (11%)	36 (15%)	186 (36%)	
Cat A	11 (20%)	50 (21%)	153 (30%)	
Cat B	14 (25%)	71 (30%)	101 (20%)	
Cat C	13 (23%)	53 (22%)	63 (12%)	
Cat D (D = demented)	0	1 (0.4%)	4 (1%)	
Cat C_D (fully dependent)	12 (21%)	30 (12%)	12 (2%)	

Data are presented as *n* (%), except for age, sex, number of comorbidities, and BMI. Nutritional status was missing in *n* = 3; results may therefore not add up to 100%

*ADL* activities of daily living, *BMI* body mass index, *MNA-SF* mini nutritional assessment—short form, *ONS* oral nutritional supplement

**Table 3:** Characteristics of the nursing home study population (*n* = 2480), by nutritional status (based on MNA-SF)

Parameter	Malnourished (*n* = 336, 14%)	At risk of malnutrition (*n* = 1214, 49%)	Normal nutritional status (*n* = 907, 37%)	*p* value
Age (years) mean ± SD	86.7 ± 6.4	86.7 ± 6.2	85.6 ± 6.2	< 0.001
Sex (% male/female)	22/78	20/80	23/77	0.258
Number of comorbidities mean ± SD	1.9 ± 1.2	1.6 ± 1.1	1.1 ± 1.0	< 0.001
BMI (kg/m^2^) ± SD	19.7 (3.9)	23.2 (4.8)	27.4 (4.7)	< 0.001
Weight loss last 3 months				< 0.001
No weight loss	34 (10%)	679 (56%)	817 (90%)	
1–3 kg	85 (25%)	307 (25%)	77 (9%)	
3–6 kg	101 (30%)	39 (3%)	0	
> 6 kg	39 (12%)	8 (1%)	0	
Unknown	77 (23%)	181 (15%)	13 (1%)	
Use of ONS	73 (22%)	111 (9%)	19 (2%)	< 0.001
Being able to climb stairs	10 (3%)	130 (11%)	246 (27%)	< 0.001
Being able to walk outside for 5 min	36 (11%)	310 (26%)	496 (55%)	< 0.001
Katz ADL score				< 0.001
Cat O	13 (4%)	133 (11%)	312 (34%)	
Cat A	13 (4%)	113 (9%)	219 (24%)	
Cat B	31 (9%)	126 (10%)	85 (9%)	
Cat C	58 (17%)	175 (14%)	92 (10%)	
Cat D (D = demented)	15 (5%)	112 (9%)	109 (12%)	
Cat C_D (fully dependent)	206 (61%)	555 (46%)	90 (10%)	

Data are presented as *n* (%), except for age, sex, number of comorbidities, and BMI. Nutritional status is missing in *n* = 23; results may therefore not add up to 100%

*ADL* activities of daily living, *BMI* body mass index, *MNA-SF* mini nutritional assessment—short form, *ONS* oral nutritional supplement

**Table 4:** Nutritional status determined with MNA-SF of community dwelling older adults and nursing home residents, by their comorbidities

	Malnourished	At risk of malnutrition	Normal nutritional status
Community dwelling (*n* = 816)	*n* = 56 (7%)	*n* = 241 (29%)	*n* = 519 (63%)
Presence of comorbidities			
Cancer (*n* = 87)	8 (9%)	33 (38%)	46 (53%)
Chronic heart failure (*n* = 113)	6 (5%)	41 (36%)	66 (58%)
COPD (*n* = 50)	8 (16%)	16 (32%)	26 (52%)
Dementia (*n* = 110)	16 (15%)	59 (54%)	35 (32%)
Depression (*n* = 44)	8 (18%)	22 (50%)	14 (32%)
Diabetes (*n* = 162)	5 (3%)	44 (27%)	113 (70%)
Fractures (*n* = 62)	4 (7%)	24 (39%)	34 (55%)
Parkinson (*n* = 37)	3 (8%)	18 (49%)	16 (43%)
Rheumatoid arthritis (*n* = 116)	5 (4%)	40 (35%)	71 (61%)
Stroke (*n* = 59)	4 (7%)	18 (31%)	37 (63%)
Nursing home (*n* = 2457)	*n* = 336 (14%)	*n* = 1214 (49%)	*n* = 907 (37%)
Presence of comorbidities			
Cancer (*n* = 169)	28 (17%)	74 (44%)	67 (40%)
Chronic heart failure (*n* = 282)	32 (11%)	150 (53%)	100 (36%)
COPD (*n* = 141)	25 (18%)	67 (48%)	49 (35%)
Dementia (*n* = 1051)	212 (20%)	649 (62%)	190 (18%)
Depression (*n* = 344)	81 (24%)	192 (56%)	71 (21%)
Diabetes (*n* = 437)	49 (11%)	208 (47%)	180 (41%)
Fractures (*n* = 310)	58 (19%)	146 (47%)	106 (34%)
Parkinson (*n* = 139)	29 (21%)	71 (51%)	39 (28%)
Rheumatoid arthritis (*n* = 122)	20 (16%)	59 (48%)	43 (35%)
Stroke (*n* = 284)	38 (13%)	147 (52%)	99 (35%)

Data are presented as *n* (%). Data represent nutritional status categorized per comorbidity (row percentage). The list of scored comorbidities was longer, reported comorbidities represent comorbidities with prevalence > 5%. Numbers do not add up because comorbidities overlap (column totals)

*COPD* chronic obstructive pulmonary disease

