# Supplementary material for: The prevalence and health burden of malnutrition in Belgian older people in the community or residing in nursing homes: results of the NutriAction II study
Source: Aging Clin Exp Res. 2018 Apr 30;31(2):175–83. doi: 10.1007/s40520-018-0957-2 (PMC6373383; doi:10.1007/s40520-018-0957-2)
Supplement: Supplementary file 1 — Supplementary material 1 (PDF 230 KB) [file 40520_2018_957_MOESM1_ESM.pdf]

Geselecteerde vragenlijst : **voedingstoestand**

NutriAction

Gegevens van de uitvoerder :
Naam en voornaam :……
rijksregisternummer………………………………………………… Geboortedatum (dd/mm/yyyy): ………………………
e-mailadres of adres (waar het resultaat van de screening naar toe mag gestuurd worden) :
……

Gebruikersgroep **uitvoerder** (kruis aan wat best past) :	**Gegevens van de patiënt**
O verpleger(ster) WZC<br>O arts WZC<br>O Wit-Gele Kruis<br>O Mederi<br>O Solidariteit voor het gezin<br>O Providhom<br>O andere verpleger(ster)<br>O andere arts<br>O andere	Nationaal registratienummer<br>………………………………………………………………………<br>Naam en voornaam………………………………………<br>………………………………………………………………………<br>Geboortedatum (dd/mm/yyyy)<br>………………………………………………………………………<br>Taal : O NL / O FR

<u>Algemene opmerkingen :</u> Gelieve de gewenste antwoorden aan te duiden of in te vullen, er zijn meerdere antwoorden per vraag mogelijk.

1. Denkt u dat de patiënt ondervoed is ☐ ja ☐ neen

2. Het geslacht van de patiënt is ☐ man ☐ vrouw

3. De leeftijd van de patiënt is ………… jaar

4. Het gewicht van de patiënt is ………… kg

Het gewicht werd o gemeten

5. De lengte van de patiënt is ………… m

6. Bovenarmomtrek van de patiënt is …………… cm (zie tekening)

☐ onbekend

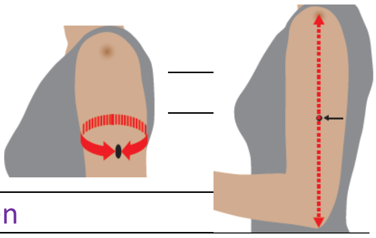

7. Denkt de patiënt dat hij/zij ondervoed is ☐ ja ☐ neen

8. Is er de laatste 3 maanden ongewenst gewichtsverlies bij de patiënt?

☐ tussen 1 en 3 kg
☐ tussen 3 en 6 kg
☐ meer dan 6 kg

☐ geen gewichtsverlies
☐ onbekend

9. Werd de patiënt de laatste 3 maanden opgenomen in het ziekenhuis?

☐ ja ☐ neen

10. Wat is de woonsituatie van de patiënt?

<u>In een instelling?</u>
☐ ziekenhuis
☐ woon- en zorgcentrum
☐ dagopvang
☐ nachtopvang

<u>Thuis</u>
☐ partner
☐ partner met externe hulp (familiehulp, thuisverpleegkundige,…)
☐ alleen
☐ alleen met externe hulp (familiehulp, thuisverpleegkundige,…)
☐ kangoeroewoning

11. Hoe zelfstandig is de patiënt in zijn/haar dagelijkse activiteit?

zich wassen
☐ zonder enige hulp
☐ gedeeltelijke hulp, boven of onder de gordel
☐ gedeeltelijke hulp zowel boven als onder de gordel
☐ volledige hulp zowel boven als onder de gordel

zich kleden
☐ zonder enige hulp
☐ gedeeltelijke hulp, boven of onder de gordel (zonder rekening te houden met veters)
☐ gedeeltelijke hulp zowel boven als onder de gordel
☐ volledige hulp zowel boven als onder de gordel

transfer en verplaatsingen
☐ volledig zelfstandig voor verplaatsing zonder mechanische hulp of hulp van derden
☐ zelfstandig voor verplaatsingen mits het gebruik van mechanische hulpmiddelen (kruk(ken),

	rolstoel, …)<br>☐ volstrekte hulp nodig van derden voor minstens één van de transfers en/of verplaatsingen<br>☐ bedlegerig of in een rolstoel en volledig afhankelijk van anderen om zich te verplaatsen
**toiletbezoek**	☐ kan alleen naar het toilet gaan, zich kleden en zich reinigen<br>☐ heeft hulp nodig voor 1 van de 3 items : zich verplaatsen of zich kleden of zich reinigen<br>☐ heeft hulp nodig voor 2 van de 3 items : zich verplaatsen en/of zich kleden en/of zich reinigen<br>☐ heeft hulp nodig voor de 3 items : zich verplaatsen en zich kleden en zich reinigen
**continentie**	☐ Is continent voor urine en faeces<br>☐ Is accidenteel incontinent voor urine of faeces (incl. blaassonde of kunstaars)<br>☐ Is incontinent voor urine (incl. mictietraining) of faeces<br>☐ Is incontinent voor urine en faeces
**eten**	☐ kan alleen eten en drinken<br>☐ heeft vooraf hulp nodig om te eten of te drinken<br>☐ heeft gedeeltelijke hulp nodig tijdens het eten of drinken<br>☐ de patiënt is volledig afhankelijk om te eten of te drinken
**tijd**	☐ geen probleem<br>☐ nu en dan, zelden probleem<br>☐ bijna elke dag probleem<br>☐ volledig gedesoriënteerd of onmogelijk te evalueren
**plaats**	☐ geen probleem<br>☐ nu en dan, zelden probleem<br>☐ bijna elke dag probleem<br>☐ volledig gedesoriënteerd of onmogelijk te evalueren

12. Welke aandoeningen zijn van toepassing voor uw patiënt ?

**Diverse:**		**Infecties:**	**Kanker:**
☐ nierinsufficiëntie met predialyse	☐ CVA	☐ luchtweginfectie	☐ borstkanker
☐ nierinsufficiëntie met dialyse	☐ fracturen	☐ urineweginfectie	☐ maagkanker
☐ diabetes	☐ COPD		☐ keelkanker
☐ depressie	☐ pre-operatief		☐ colonkanker
☐ dementie	☐ post-operatief		☐ longkanker
☐ Parkinson	☐ reumatoïde artritis		☐ prostaatkanker
	☐ chronisch hartfalen		☐ andere

13. Welke bijkomende problemen vertoont uw patiënt?

☐ oedeem
☐ slikproblemen
☐ doorligwonden
☐ kauwproblemen
☐ ernstige stress door ziekte en/of psychologische problemen tijdens de laatste 3 maanden.

14. Is de patiënt minder gaan eten tijdens de laatste 3 maanden ?

☐ niet minder gaan eten — ☐ veel minder gaan eten
☐ matig minder gaan eten — ☐ geen voedselinname mogelijk de laatste > 5 dagen

15. Hoeveel verschillende medicamenten neemt de patiënt gemiddeld per dag?

gemiddeld ……….. verschillende medicamenten
☐ onbekend

16. Hoeveel maaltijden gebruikt de patiënt per dag? (met inbegrip van tussendoortjes)

gemiddeld ……….. maaltijden
☐ onbekend

17. Gebruikt de patiënt medische bijvoedingen (rijk aan energie en/of eiwitten)?

☐ ja — ☐ neen
Per sonde ? ☐ ja ☐ neen

18. Kan de patiënt een trap van 15 treden of meer op- en aflopen zonder te rusten?

☐ ja — ☐ neen

19. Kan de patiënt 5 minuten buiten wandelen zonder te rusten?

☐ ja — ☐ neen

20. Welke schaal wenst u te gebruiken voor de berekening?

☐ MUST — ☐ MNA®- short

Bedankt voor het invullen van de vragenlijst.
