# Supplementary material for: The prevalence and health burden of malnutrition in Belgian older people in the community or residing in nursing homes: results of the NutriAction II study
Source: Aging Clin Exp Res. 2018 Apr 30;31(2):175–83. doi: 10.1007/s40520-018-0957-2 (PMC6373383; doi:10.1007/s40520-018-0957-2)
Supplement: Supplementary file 2 — Supplementary material 2 (DOCX 31 KB) [file 40520_2018_957_MOESM2_ESM.docx]

**The prevalence and health burden of malnutrition in Belgian older people in the community or residing in nursing homes: results of the NutriAction II study**

Maurits F.J. Vandewoude^1^, Janneke P. van Wijngaarden^2^, Lieven De Maesschalck^3^, Yvette C. Luiking^2^, André Van Gossum^4^

^1^Department of Geriatrics (ZNA), University of Antwerp, Antwerp, Belgium

^2^Nutricia Research, Nutricia Advanced Medical Nutrition, Utrecht, the Netherlands

^3^Mobilab, Thomas More University college, Geel, Belgium

^4^Department of Gastroenterology, Nutrition Support Team, Hôpital Erasme,ULB, Brussels, Belgium

***Corresponding author and person to whom reprint requests should be addressed****:*

Maurits Vandewoude, Department of Geriatrics (ZNA), University of Antwerp, Antwerp, Belgium, e-mail: [maurits.vandewoude@zna.be](mailto:maurits.vandewoude@zna.be), telephone +32-3-234 42 49

**Supplemental Table 1a: Comorbidities and ADL in community dwelling older adults (n=819)**

|  | **Katz O**  n= 228 (28%) | **Katz A**  n= 214 (26%) | **Katz B**  n= 188 (23%) | **Katz C**  N= 129 (16%) | **Katz D**  n=5 (0.6%) | **Katz C_D**  n =55 (7%) |
| --- | --- | --- | --- | --- | --- | --- |
| Presence of comorbidities   - Cancer (n=87) - Chronic heart failure (n=113) - COPD (n=50) - Dementia (n=113) - Depression (n=47) - Diabetes (n=163) - Fractures (n=63) - Parkinson (n=37) - Rheumatoid arthritis (n=116) - Stroke (n=59) | 24 (28%)  24 (21%)  9 (18%)  11 (10%)  7 (15%)  58 (36%)  11 (18%)  4 (11%)  22 (19%)  9 (15%) | 32 (37%)  36 (32%)  12 (24%)  11 (10%)  13 (28%)  35 (22%)  20 (32%)  11 (30%)  39 (34%)  12 (20%) | 14 (16%)  26 (23%)  20 (40%)  25 (22%)  15 (32%)  37 (23%)  22 (35%)  10 (27%)  31 (27%)  14 (24%) | 16 (18%)  19 (17%)  9 (18%)  17 (15%)  6 (13%)  18 (11%)  9 (14%)  8 (22%)  15 (13%)  19 (32%) | 0  1 (1%)  0  4 (4%)  1 (2%)  1 (1%)  0  0  1 (1%)  1 (2%) | 1 (1%)  7 (6%)  0  45 (40%)  5 (11%)  14 (9%)  1 (2%)  4 (11%)  8 (7%)  4 (7%) |

Data are presented as n (%). ADL, Activities of Daily Living; COPD, chronic obstructive pulmonary disease; Katz D, Katz Demented

**Supplemental Table 1b: Comorbidities and ADL in nursing home residents (n=2480)**

|  | **Katz O**  n = 460 (19%) | **Katz A**  n = 346 (14%) | **Katz B**  n = 242 (10%) | **Katz C**  n = 327 (13%) | **Katz D**  n = 242 (10%) | **Katz C_D**  n = 863 (35%) |
| --- | --- | --- | --- | --- | --- | --- |
| Presence of comorbidities   - Cancer (n=171) - Chronic heart failure (n=285) - COPD (n=141) - Dementia (n=1074) - Depression (n=367) - Diabetes (n=440) - Fractures (n=313) - Parkinson (n=140) - Rheumatoid arthritis (n=122) - Stroke (n=284) | 36 (21%)  46 (16%)  22 (16%)  51 (5%)  74 (20%)  67 (15%)  53 (17%)  14 (10%)  17 (14%)  26 (9%) | 35 (21%)  41 (14%)  22 (16%)  67 (6%)  56 (15%)  66 (15%)  52 (17%)  9 (6%)  17 (14%)  31 (11%) | 12 (7%)  45 (16%)  20 (14%)  33 (3%)  38 (10%)  48 (11%)  30 (10%)  26 (19%)  20 (16%)  25 (9%) | 26 (15%)  38 (13%)  25 (18%)  59 (6%)  54 (15%)  62 (14%)  42 (13%)  23 (16%)  26 (21%)  82 (29%) | 13 (8%)  16 (6%)  10 (7%)  191(18%)  31 (8%)  45 (10%)  23 (7%)  3 (2%)  9 (7%)  15 (5%) | 49 (29%)  99 (35%)  42 (30%)  673 (63%)  114 (31%)  152 (35%)  113 (36%)  65 (46%)  33 (27%)  105 (37%) |

Data are presented as n (%). ADL, Activities of Daily Living; COPD, chronic obstructive pulmonary disease; Katz D, Katz Demented
